# Neurofilament light chains in serum as biomarkers of axonal damage in early MS lesions: a histological–serological correlative study

**DOI:** 10.1007/s00415-022-11468-2

**Published:** 2022-11-13

**Authors:** Anne-Sophie Dietmann, Niels Kruse, Lidia Stork, Mareike Gloth, Wolfgang Brück, Imke Metz

**Affiliations:** Institute of Neuropathology, University Medical Center Göttingen, Robert-Koch-Strasse 40, 37075 Göttingen, Germany

**Keywords:** Neurofilament light chains, Axonal damage, Early multiple sclerosis

## Abstract

Multiple sclerosis (MS) is a chronic inflammatory demyelinating disease associated with axonal injury, and neurofilament light chains in serum (sNfL) are considered a biomarker for this damage. We aimed to investigate the relationship between sNfL and the axonal damage in early MS lesions in a special cohort of biopsied patients. sNfL from 106 biopsied patients with 26 follow-up samples were analyzed using single-molecule array (SiMoA) technology. Findings were correlated with clinical parameters and histological findings of acute axonal damage (APP-positive spheroids) and axonal loss in different lesion stages. A median of 59 pg/ml sNfL was found (range 8–3101 pg/ml). sNfL levels correlated with APP-positive spheroids in early active demyelinating lesions that represent the earliest lesion stages (*p* < 0.01). A significant negative correlation between sNfL levels in follow-up blood samples and axonal density in normal-appearing white matter was also observed (*p* = 0.02). sNfL levels correlated with the Expanded Disability Status Score at biopsy (*p* < 0.01, *r* = 0.49) and at last clinical follow-up (*p* < 0.01, *r* = 0.66). In conclusion, sNfL likely represent a compound measure of recent and ongoing neuroaxonal damage. We found that sNfL in biopsied MS patients correlate with acute axonal damage in the earliest MS lesion stages. Determination of sNfL levels thus allows insight into brain pathology and underlines the relevance of relapse-associated lesional pathology. Axonal loss in normal-appearing white matter contributes to sNfL levels independent of relapses. Since sNfL levels correlate with clinical disability, they may predict the future disability of patients and help with individual treatment decisions.

**Supplementary Information:**

The online version contains supplementary material available at 10.1007/s00415-022-11468-2.

## Introduction

Multiple sclerosis (MS) is an inflammatory demyelinating disease of the CNS that is associated with varying degrees of axonal damage. This damage is most pronounced in white matter lesions, but may also occur in the non-demyelinated, so-called normal-appearing white matter (NAWM) as well as in the grey matter [5, 21]. Axonal damage is considered acute when it has occurred in the last weeks to months prior to its detection in biopsy material using immunohistochemical staining for amyloid precursor protein (APP) [17]. Previous investigations showed that acute axonal damage within lesions is most prominent in early disease stages [29]. However, axonal damage accumulates, leading to pronounced axonal loss that often exceeds 50% in chronic MS lesions [35, 38, 44]. Changes in the normal-appearing white and grey matter lesions are more prevalent in chronic MS [30].

Axonal damage is irreversible, since no relevant neuroaxonal regeneration is observed within the CNS [11, 19, 24, 37, 47]. Therefore, it is of utmost importance to monitor and hinder axonal damage from the earliest disease stages on.

Recent findings indicate that changes in serum neurofilament light chain (sNfL) concentration may serve as promising biomarker for axonal damage. NfL are structural scaffolding protein components of the cytoskeleton that are exclusively present in neurons and axons, and their detection in CSF or serum thus specifically indicates neuroaxonal damage [3, 23, 36]. sNfL correlate with NfL levels in CSF, and although NfL concentrations are low in serum, reliable measurement can be achieved with ultrasensitive methods such as the single-molecule array (SiMoA) technique [26]. Numerous studies on sNfL levels in MS patients have shown that sNfL are biomarkers of disease activity and indicate disease prognosis as well as treatment responses [1, 14, 18, 27, 28, 42, 45].

Detection of sNfL does not, however, provide any information on the localization of the neuroaxonal damage [14]. Furthermore, multiple factors, e.g., patient age, can also influence sNfL levels [14, 28]. Thus far, no histopathological-serological studies have been performed in early MS to show the pathological correlate of elevated sNfL levels. Therefore, we aimed to provide a detailed characterization of the acute and chronic axonal damage in MS lesions and adjacent non-demyelinated normal-appearing white matter from biopsied patients, and correlated our findings with the sNfL levels from these patients.

## Materials and methods

### Study cohort

This cohort study was approved by the ethics committee of the University Medical Center Göttingen (#19/09/10) and informed consent was obtained from each patient. The study has therefore been performed in accordance with the ethical standards laid down in the 1964 Declaration of Helsinki and its later amendments. The study included analysis of formalin-fixed, paraffin-embedded archival brain tissue and serum samples from 106 subjects with brain-biopsy inflammatory demyelination consistent with MS. Brain biopsies were performed for differential diagnostics (e.g., to rule out a tumor) and not for research purposes. After the biopsy revealed pathological changes compatible with MS, patients were included in the MS Lesion Project, a retrospective as well as prospective clinical study to analyze pathologic, clinical, serological, and radiographic correlates of MS lesions. Inclusion criteria for the present study were: (1) histopathological diagnosis of inflammatory demyelinating lesions consistent with MS, (2) no other confounding pathology, and (3) available serum for determining sNfL levels. We excluded patients with related diseases such as neuromyelitis optica spectrum diseases (NMOSD), defined by clinical, serological, and histological criteria [8, 40, 49] or acute disseminated encephalomyelitis (ADEM), defined as demyelination limited to perivenular areas [9].

### Neuropathological analyses

Formalin-fixed, paraffin-embedded slides were stained with hematoxylin and eosin, luxol fast blue/periodic acid-Schiff and Bielschowsky’s silver impregnation. Immunohistochemistry was performed with antibodies against myelin proteins [anti-myelin basic protein (MBP), anti-proteolipid protein (PLP), anti-myelin oligodendrocyte glycoprotein (MOG), anti-2′3′-cylic nucleotide 3′phosphodiesterase (CNP), and anti-myelin-associated glycoprotein (MAG)] as well as the acute axonal damage marker amyloid precursor protein (APP; MAB348, Clone 22C11, Millipore). Detailed information has been previously published by Stork et al. [43].

**Fig. 1:**
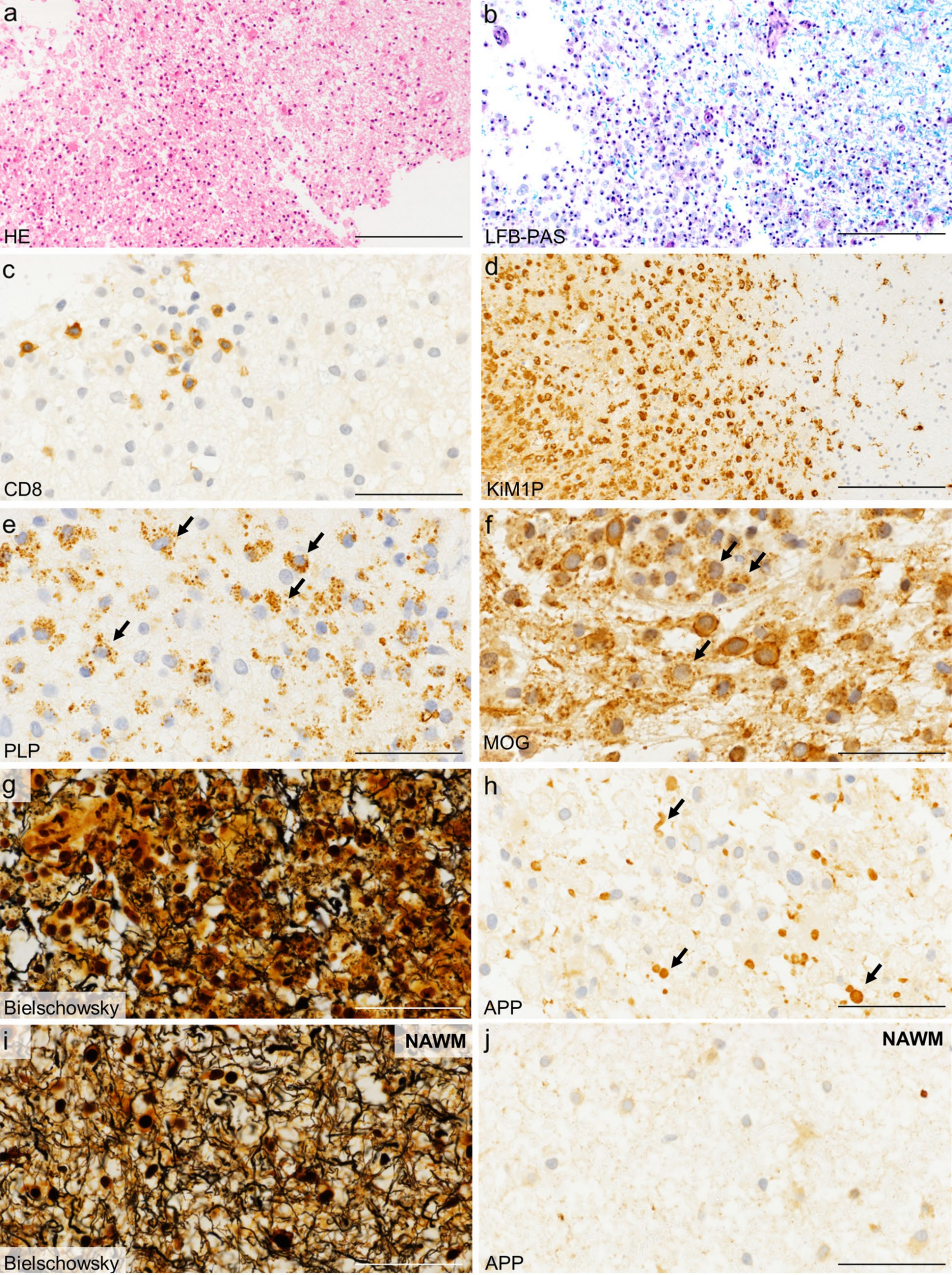
Histology of biopsied MS lesions. HE shows cellular white matter lesions (**a**) with demyelination (**b**, LFB/PAS), as indicated by the missing blue myelin staining. The inflammatory infiltrate consists of T cells (**c**, anti-CD8 staining for cytotoxic T cells) and numerous macrophages (**d**, KiM1P staining). Myelin degradation products within macrophages allow a staging of the demyelinating activity. Major myelin proteins (**e**, anti-PLP) and minor myelin proteins (**f**, anti-MOG) are present within macrophages in this example, indicating an early active demyelinating lesion. The axonal density can be determined with Bielschowsky silver staining (**g**, **i**) that shows a more pronounced axonal reduction within lesions (**g**) compared to the normal-appearing white matter (**i**). Also, more acutely damaged axons are found within lesions (**h**, anti-APP) compared to the normal-appearing white matter (**j**, anti-APP). *HE* hematoxylin/eosin, *LFB/PAS* luxol fast blue/periodic acid-Schiff, *PLP* proteolipid protein, *MOG* myelin oligodendrocyte glycoprotein, *APP* amyloid precursor protein, *NAWM* normal-appearing white matter. Scale bar **a**, **b** and **d** 200 µm; **c**, **e**–**j** 50 µm

In a first step, the demyelinating activity was determined according to published criteria: Early active demyelinating lesions (EA lesions) were defined by myelin-laden macrophages immunoreactive both for minor (CNP, MOG, MAG) and major (MBP, PLP) myelin proteins (Fig. 1); late active lesions (LA) are immunoreactive for major myelin proteins, but not minor myelin proteins. Inactive lesions (IA) show no further immunoreactivity within myelin-laden macrophages, neither for major nor for minor myelin proteins [15]. These lesion stages indicate the age of demyelinating lesions, with early active demyelinating lesions representing the earliest stages of lesion formation. We defined as periplaque white matter (PPWM) the non-demyelinated region of two microscopic fields (× 40) adjacent to demyelinated areas and as normal-appearing white matter (NAWM) non-demyelinated regions more distant to plaques. More than one lesion area (demyelinating activity) can be present in a single biopsy.

In a next step, we determined the acute axonal damage with the anti-APP staining and the relative axonal density by staining with Bielschowsky’s silver impregnation in the respective lesion areas (Fig. 1). The number of APP-positive axons was counted in at least three standardized microscopic fields of 10,000 μm^2^, each defined by an ocular morphometric grid, and data are given as APP-positive spheroids per mm^2^. The relative axonal density (which we refer to here for simplicity as axonal density) was determined by point sampling using a 25-point Zeiss eyepiece. The axonal density was expressed as the number of axons crossing the stereological grid points from the total number of 25 grid points. Measurements were performed using a field size of 95,000 μm^2^, and again, at least three microscopic fields were evaluated. The median value of APP-positive spheroids as well as the median axonal density in each patient were used for further statistical analyses.

### Clinical assessment

Clinical information was obtained via medical record review as well as a personal interview and examination. The Expanded Disability Status Scale (EDSS) score was determined at different time points: EDSS at biopsy, EDSS at the timepoint of the first and second blood sampling, and EDSS at last follow-up. The clinical course was defined according to published criteria into monophasic, relapsing–remitting, progressive relapsing and primary or secondary progressive [46]. In addition, we recorded relapses as well as a relapse therapy within 6 months and different therapy modalities within 6 weeks prior to dates of blood sampling. A relatively long interval of 6 months was chosen for relapses, as study visits with blood sampling were usually not feasible within 6 weeks after patients suffered from relapses. However, an effect of relapses on sNfL levels was still likely within a longer time interval. Therapies were classified as immunomodulating/immunosuppressive (interferonß-1a, interferonß-1b, glatiramer acetate, fingolimod, dimethyl fumarate, azathioprine, rituximab, mitoxantrone, cyclophosphamide), relapse therapy (either high-dose corticosteroids or apheresis treatments) and, to analyze a specific steroid effect, as steroid treatment (either low-dose or high-dose steroids). The time intervals between symptom onset and biopsy and between biopsy and blood sampling, as well as last follow-up examination, were noted.

### Blood sampling

Blood sampling was done at baseline for all 106 patients at variable timepoints relative to the biopsy (median 9.6 months after biopsy, range -0.1–198 months). The time interval from biopsy to baseline blood sampling is shown in Online resource 1. For a subset of patients (*n* = 26), a second blood sampling was performed to assess the change over time of sNfL levels (median 20.8 months after biopsy, range (7.4–33.2 months). From 10 healthy controls (median age 50.5 years; 5 males, 5 females), blood samples were taken and sNfL levels measured to obtain a range for normal values.

### sNfL measurement with SiMoA technology

Serum samples were stored at − 20 °C and − 80 °C until analysis. Quantification of neurofilament light chains (NfL) was done using the SiMoA NF-light Advantage Kit (Quanterix, Bellerica, MA, USA; Cat. No. 103186). Assays were performed on a SiMoA HD-1 Analyzer (Quanterix). Serum aliquots (100–200 µl) and calibrators of predefined concentration were thawed at room temperature. Serum samples were centrifuged for 5 min at 10,000*g* and diluted 1 in 4 with sample diluent. Diluted samples and calibrators were transferred to Quanterix microtiter plates and sealed with X-Pierce™ sealing film to prevent evaporation. The analytical procedure was set up by scanning the barcodes of the kit components [bead reagent, detector reagent, streptavidin-ß-galactosidase (SBG) reagent, resorufin ß-d-galactopyranoside (RGP) reagent] before placing them in the instrument. The assay was performed according to a Quanterix-developed protocol in the HD-1 Analyzer. After completion of the assay, data were analyzed using Quanterix software.

### Statistical analyses

Statistical analyses were done using the Mann–Whitney *U* test and subsequent Bonferroni correction to account for multiple comparisons. Simple linear regression analyses as well as multiple linear regression analyses were performed. Simple linear regression analyses were chosen as the group size was limited and not always suitable for multiple regression analyses. Multiple linear regression analyses were done to account for multiple factors influencing the sNfL at the same time. For multiple linear regression analyses, a logarithmic transformation of sNfL levels was carried out. To compare sNfL levels from the first and second blood sampling, the Wilcoxon test was used. *Graphpad Prism 6* and *SSPS Statistics 25 for Windows* were taken for statistical analyses. Tests were classified as significant if the *p* value was < 0.05.

## Results

### Patient demographics

Patient demographic data at biopsy as well as first and second blood sampling are shown in Table 1. At biopsy, 76 patients had a monophasic disease course, 27 a relapsing–remitting, and one patient a progressive relapsing course. Data were not available for two patients. At last follow-up at a median time of 2.48 years after biopsy (range 0.02–17.47 years), 44 patients had a monophasic disease course, 46 patients a relapsing–remitting course, one patient a progressive relapsing, and eight patients a secondary progressive course. No data were available for eight patients.

**Table 1:** Descriptive statistics of demographic and clinical variables of patients at biopsy, first and second blood sampling

	MS patients		
	At biopsy (*n* = 106)	First blood sampling (*n* = 106)	Second blood sampling (*n* = 26)
Age in years, mean (min–max)	39.77 (9.46–85.73)	41.20 (11.21 – 86.67)	42.37 (15.45–65.23)
Gender, m:f (%)	47:59 (44:56)		17:9 (65:35)
Time interval biopsy to blood sampling in months, median (min–max)	–	9.57 (− 0.1–198.03)	20.83 (7.37–33.17)
Time interval first clinical symptoms to biopsy in days, median (min–max)	34 (3–11,778)		
EDSS score, median	4	3	3
Relapse therapy 6 weeks before blood sampling, yes:no	–	5:100 n.a.: 1	1:25
Immunomodulating or -suppressive therapies 6 weeks before blood sampling, yes:no	–	30:73 n.a.: 3	10:16
Steroids 6 weeks before blood sampling, yes:no	–	9:96 n.a.: 1	1:25
Relapse within 6 months before blood sampling, yes:no		22:81 n.a.: 3	1:19 n.a.: 6

*min* minimum, *max* maximum, *m* male, *f* female, *n.a.* not available, *EDSS* Expanded Disability Status Scale

### Axonal damage shown by histology

All patients showed pathological features typical for MS with confluent inflammatory demyelinating lesions and a relative axonal sparing (Fig. 1). A relative axonal sparing means that a variable number of axons are damaged, but not all axons. At first, we determined the demyelinating lesion activities of the 106 biopsies and identified 87 early active demyelinating (EA), 31 late active demyelinating (LA), and 22 inactive demyelinated lesion areas (IA), as well as 69 periplaque white matter (PPWM) and 58 normal-appearing white matter lesion areas (NAWM). Lesion staging is essential, as the acute axonal damage and axonal density vary in different lesion stages. Next, we determined acute axonal damage with anti-APP staining and axonal density with Bielschowsky silver impregnation in these different lesion areas.

#### Acute axonal damage in MS lesions and non-demyelinated white matter

**Fig. 2:**
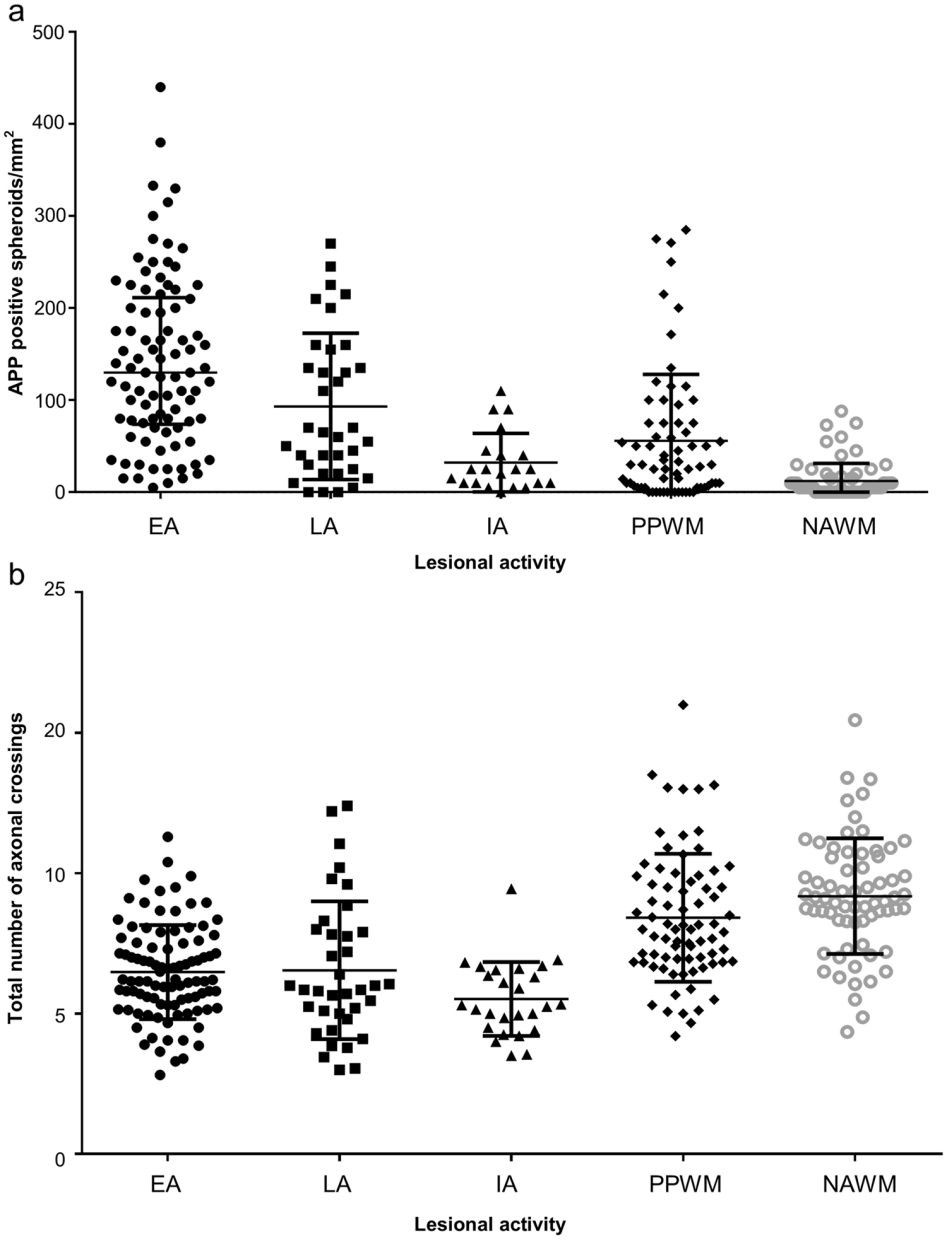
Axonal damage in different lesion areas. Acute axonal damage (**a**) and axonal density (**b**) in different lesion areas are shown. Acute axonal damage was determined by the number of APP-positive spheroids (**a**), and axonal density by counting the axonal crossings using a microscopic grid and Bielschowsky’s silver staining (**b**). *EA* early active demyelinating, *LA* late active demyelinating, *IA* inactive demyelinated, *PPWM* periplaque white matter, *NAWM* normal-appearing white matter, *APP* amyloid precursor protein

The number of APP-positive axonal spheroids per mm^2^ in different lesion areas is shown in Fig. 2a. The highest number of spheroids was found in early active demyelinating lesions (median 130 spheroids/mm^2^), consistent with previous studies [15, 17, 25]. Significantly more spheroids were found in lesion areas (EA, LA, IA) and the PPWM compared to the NAWM (median: EA 130 spheroids/mm^2^; LA 65/mm^2^; IA 25/mm^2^; PPWM 30/mm^2^; NAWM 5/mm^2^). With increasing lesion age, the number of acutely damaged axons was observed to decrease; this was seen by comparing EA, LA and IA lesion areas.

#### Axonal density in MS lesions and non-demyelinated white matter

The same lesion areas were analyzed for their axonal density (Fig. 2b). Lesion areas showed a significantly lower axonal density compared to the normal-appearing white matter, with inactive lesions having the lowest numbers (median 5.28 crossing axons/25 grid’s points). Early active and late active lesions showed comparable axonal densities (median EA 6.2 axons/25 points und LA 5.85 axons/25 points). In the periplaque white matter, axonal density was also reduced compared to the normal-appearing white matter (median PPWM 8 axons/25 points und NAWM 9.05 axons/25 points), although numbers were higher than in lesion areas.

No differences with respect to gender or age of patients were found for either acute axonal damage or axonal density (data not shown).

### Neurofilament light chains in serum (sNfL) in a biopsied cohort of MS patients

Blood samples were taken from biopsied MS patients with histologic quantification of axonal damage. They were taken at varying time intervals after the biopsy, with a median of 9.6 months for the first blood sampling (baseline) and a median of 20.8 months for the second blood sampling (follow-up, see also Table 1).

**Fig. 3:**
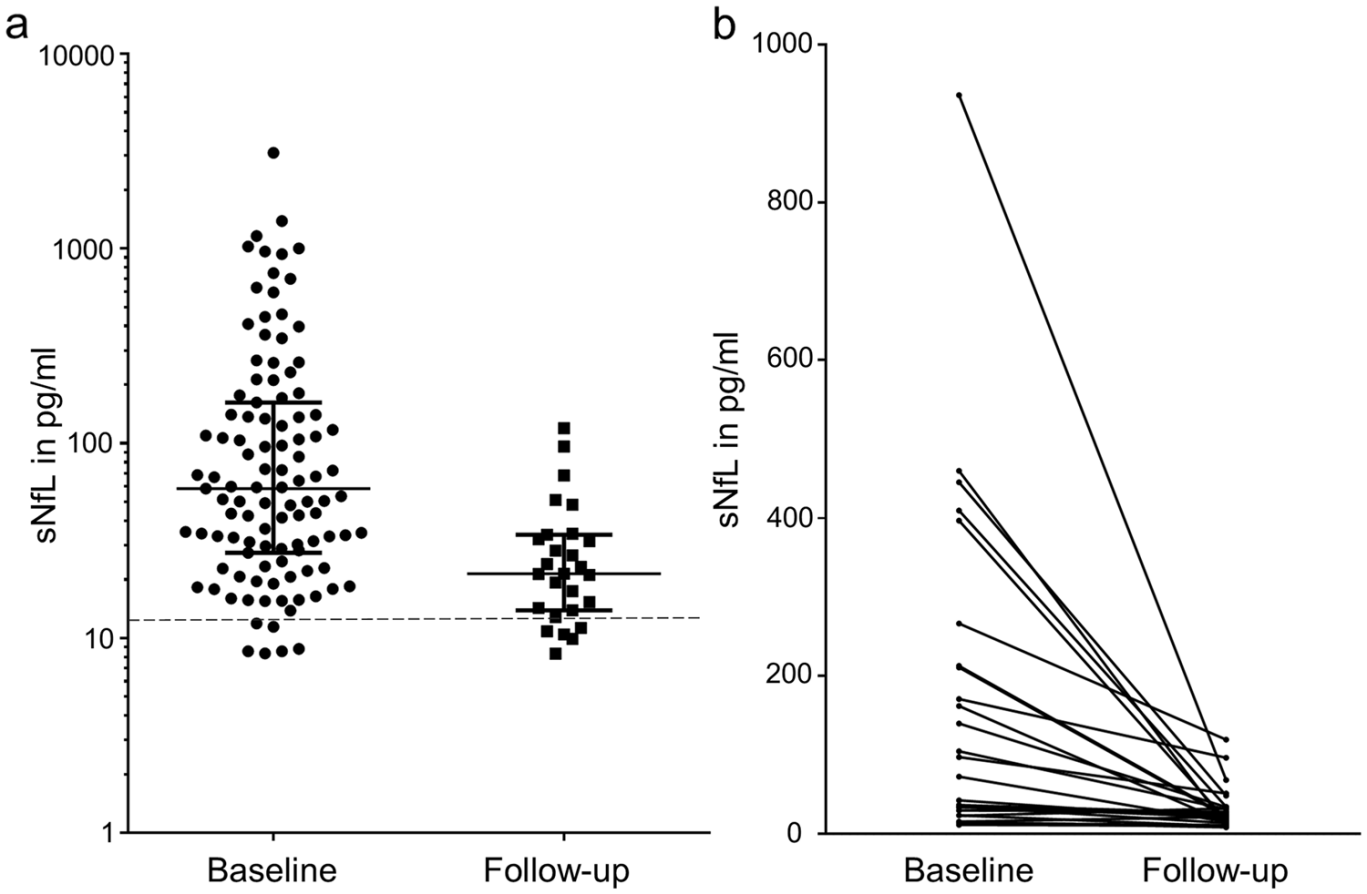
sNfL levels in biopsied MS patients at baseline and follow-up. **a** sNfL levels at baseline (median 58.95 pg/ml) are significantly higher than at follow-up (median 21.34 pg/ml). The median, 25th as well as 75th percentiles are shown. Healthy controls (*n* = 10) are represented by the dashed line (median 11.03 pg/ml). **b** sNfL levels of patients with two consecutive blood samplings are shown with the two levels of individual patients connected by a line (median baseline: 84.96 pg/ml, median follow-up: 22.29 pg/ml). sNfL levels decrease in all patients, except for two patients with initial low levels and minor increases over time

Very high sNfL levels were found at baseline (median baseline: 58.95 pg/ml; median healthy controls: 11.03 pg/ml, *p* < 0.0001, Fig. 3a). The highest sNfL level with 3101.15 pg/ml was found in a 14.5-year-old child with a fulminant MS disease course in a blood sample taken one month after biopsy. At follow-up, significantly lower sNfL levels were detectable in biopsied MS patients compared to baseline (median: 21.34 pg/ml, *p* < 0.0001), but sNfL were still significantly higher than in healthy controls (*p* < 0.001).

Comparing baseline and follow-up sNfl levels in our cohort with consecutive samples (*n* = 26 patients), 24 of those patients showed a decrease in sNfL levels over time (Fig. 3b). Only two patients with low sNfL levels at baseline showed minor increases, possibly reflecting natural fluctuations.

### Epidemiological and clinical characteristics that affect sNfL levels

Simple and multiple linear regression analyses as well as group comparisons were done to investigate epidemiological and clinical characteristics that correlate with sNfL.

#### Age, but not gender, correlates with sNfL levels

Simple linear regression analyses of baseline and follow-up samples (*n* = 132) did not provide a correlation of sNfL levels with age, although this was shown in prior publications [1, 14, 28, 31]. However, in multiple linear regression analyses, a correlation with age could be found (see model to explain sNfL levels below; *p* < 0.01). No significant differences in sNfL levels were found between male and female patients.

#### With a longer time interval between biopsy and blood sampling, sNfL levels decrease

Simple as well as multiple linear regression analyses revealed a negative correlation between sNfL levels of the baseline and follow-up blood samples and the time that passed after biopsy (*p* = 0.01 for simple linear regression analysis).

#### Higher sNfL levels are found when relapses occurred prior to blood sampling

Comparing sNfL levels from patients with and without a relapse within 6 months prior to baseline blood sampling showed that patients with relapses had significantly higher sNfL levels (*p* < 0.001). However, some patients without a clinically evident relapse also showed high sNfL levels (Online Resource 2a). Multiple linear regression analyses confirmed these findings.

#### Therapies given prior to blood sampling affect sNfL levels

We investigated sNfL levels in baseline and follow-up samples from patients with and without (1) relapse therapies, (2) immunomodulating/immunosuppressive therapies, and (3) steroid treatments within 6 weeks prior to blood sampling (see also method section). Group comparisons showed that patients with a relapse therapy had higher sNfL levels than patients without a relapse therapy (*p* < 0.001). Group comparisons did not show differences in patients treated with immunomodulatory/immunosuppressive therapies compared to patients without such treatments, but multiple linear regression analyses did show higher sNfL levels in patients treated with immunomodulatory/immunosuppressive therapies (see model to explain sNfL levels below). Group comparisons in addition indicated that patients who received a steroid treatment (either high- or low-dose corticosteroids) had higher sNfL levels than patients without such treatment (*p* < 0.01, Online Resource 2b). These results were confirmed by multiple linear regression analyses.

#### Open surgery versus stereotactic biopsy does not influence sNfL levels

We hypothesized that open surgery might cause more pronounced tissue damage and accordingly higher sNfL levels than a stereotactic biopsy, and thus compared sNfL levels of baseline blood samples of those two groups (30 patients who underwent open surgery, 72 patients with a stereotactic biopsy, 4 patients with no information available). However, no significant differences in sNfL levels were found (*p* = 0.71).

### Correlation of the axonal damage as shown by histology and neurofilament light chains in serum

Next, we investigated whether histologically proven axonal damage of different lesion stages could be correlated with sNfL levels. Demyelinating lesional activities as well as periplaque and normal-appearing white matter show varying degrees of axonal damage and thus may have varying effects on sNfL levels. Baseline as well as follow-up blood samples were also assessed separately. Furthermore, we analyzed correlations of acute axonal damage as shown with the anti-APP staining and axonal density as determined by Bielschowsky silver staining. Simple linear regression analyses as well as multiple linear regression were performed.

#### sNfL correlate with the acute axonal damage in early active demyelinating MS lesions

In simple linear regression analyses, no correlations between the number of APP-positive spheroids in different demyelinating lesions areas (early active, late active or inactive lesions), the periplaque white matter or the normal-appearing white matter, and sNfL levels were found. In this analysis, other factors that influence sNfL levels (such as the age of patients or prior relapses) were not considered.

In contrast, in multiple linear regression models, a significant correlation between the number of APP-positive spheroids in early active demyelinating lesions and sNfL levels in baseline blood samples was found (see model to explain sNfL levels below; *p* < 0.01). This indicates that the acute axonal damage in the earliest lesion stages contributes to sNfL levels. No correlations were found between sNfL levels and acute axonal damage as shown by histology in late active and inactive lesions, the periplaque white matter or normal-appearing white matter.

#### sNfL levels inversely correlate with axonal density in the normal-appearing white matter of follow-up samples

Simple regression models correlating sNfL levels with axonal density revealed a significant inverse correlation between the sNfL levels of the follow-up blood samples (median 21 months after the biopsy) and axonal density in normal-appearing white matter (*n* = 16 patients in this subgroup, *p* = 0.02; Online Resource 3). When axonal density was lower in normal-appearing white matter, higher sNfL levels were detected. No further significant correlations were found.

However, multiple regression analyses could not confirm these results, as the sample number was too small (only *n* = 9 patients could be analyzed as only those had all data available that were needed for multiple regression analysis). This group was also too small to perform bootstrapping, a statistical procedure that resamples the dataset to simulate more samples.

### Approximation of a model to explain sNfL levels

Multiple regression analysis, as discussed above, was performed to analyze which factors had a simultaneous impact on sNfL levels, and the data were used to develop a model to explain sNfL levels. Variables that were initially included in this model were: APP-positive spheroids in early active lesions, time interval between biopsy and first blood sampling in months, age (years) at the time of first blood sampling, gender, type of biopsy, immunomodulating/immunosuppressive treatment 6 weeks prior to blood sampling, steroid treatment 6 weeks prior to blood sampling, relapses within 6 months prior to first blood sampling and relapse therapy 6 months prior to first blood sampling. Variables with no significant effect on sNfL levels were excluded one after the other, until only variables with a significant effect on sNfL levels were left. For this model, only sNfL levels from baseline blood samples were included.

Variables that turned out to be significantly correlated with sNfL, as mentioned above, included histologically determined acute axonal damage (APP-positive spheroids) in early active demyelinating lesions. Demographic and clinical data that impacted sNfL levels were: (1) age (older patients had higher sNfl levels); (2) time interval between biopsy and blood sampling (with longer time, sNfL levels decreased); (3) relapses within six months prior to blood sampling (with higher sNfL levels when relapses occurred); and (4) treatment with steroids and/or immunomodulating/immunosuppressive therapies within 6 weeks prior to blood sampling (patients with such treatment had higher sNfL levels). The model is summarized in Table 2. The corrected *R*^2^ for this model was 45.5%, meaning that 45.5% of the variance of sNfL levels are explained with this model.

**Table 2:** Model to explain sNfL levels in biopsied MS patients

*N* = 78	Unstandardized coefficient	Standardized coefficient	Significance	95% Confidence interval Lower and upper bound	
APP-positive spheroids in early active demyelinating lesions	< 0.01	0.23	< 0.01	0.00	< 0.01
Age (years) at baseline blood sampling	0.02	< 0.01	< 0.01	< 0.01	0.2
Time interval (months) between biopsy and baseline blood sampling	< − 0.01	− 0.2	< 0.01	< − 0.01	0.00
Relapse within 6 months prior to baseline blood sampling	0.42	0.28	< 0.01	0.12	0.8
Steroid treatment within 6 weeks prior to baseline blood sampling	0.72	0.34	< 0.01	0.27	1.18
Immunomodulating/immunosuppressive therapy within 6 weeks prior to baseline blood sampling	0.28	0.21	0.02	0.04	0.52

sNfL level at baseline was defined as dependent variable. Shown are all variables with a significant effect on sNfL levels, including the unstandardized coefficient, the standardized coefficient, the significance, and the confidence interval. This model has a corrected *R*^2^ = 45.5%

**Fig. 4:**
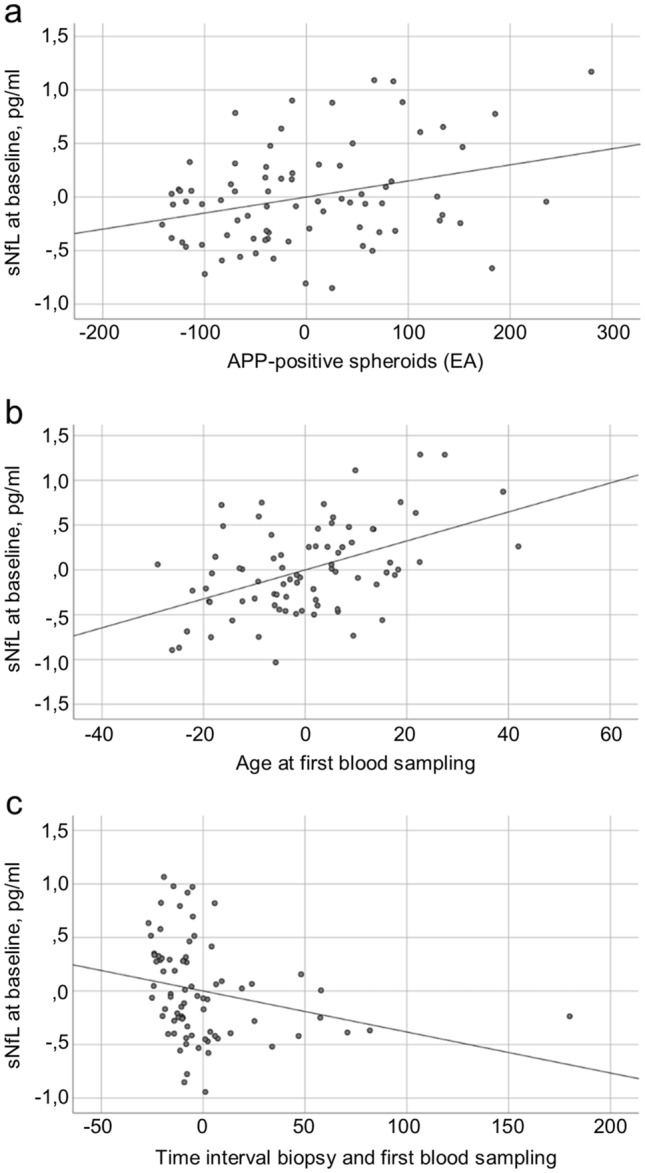
Partial regression plots of variables that contribute to sNfL levels. The dependent variable is the sNfL level at baseline. Independent variables are in **a** APP-positive spheroids in early active demyelinating MS lesions, **b** age at baseline, and **c** the time interval between biopsy and baseline. Due to calculations with multiple variables, the scaling of the axes differs from normal units and does not seem plausible. **a** sNfL levels correlate positively with the APP-positive spheroids in early active demyelinating (EA) lesions (*p* < 0.01). **b** sNfL levels increase with age (*p* < 0.01). **c** sNfL levels correlate negatively with the time interval between biopsy and blood sampling (*p* < 0.01)

Figure 4 shows partial regression plots of those continuous variables with a significant effect on sNfL levels, calculated from the above multiple linear regression analysis.

### sNfL levels correlate with clinical disability

**Fig. 5:**
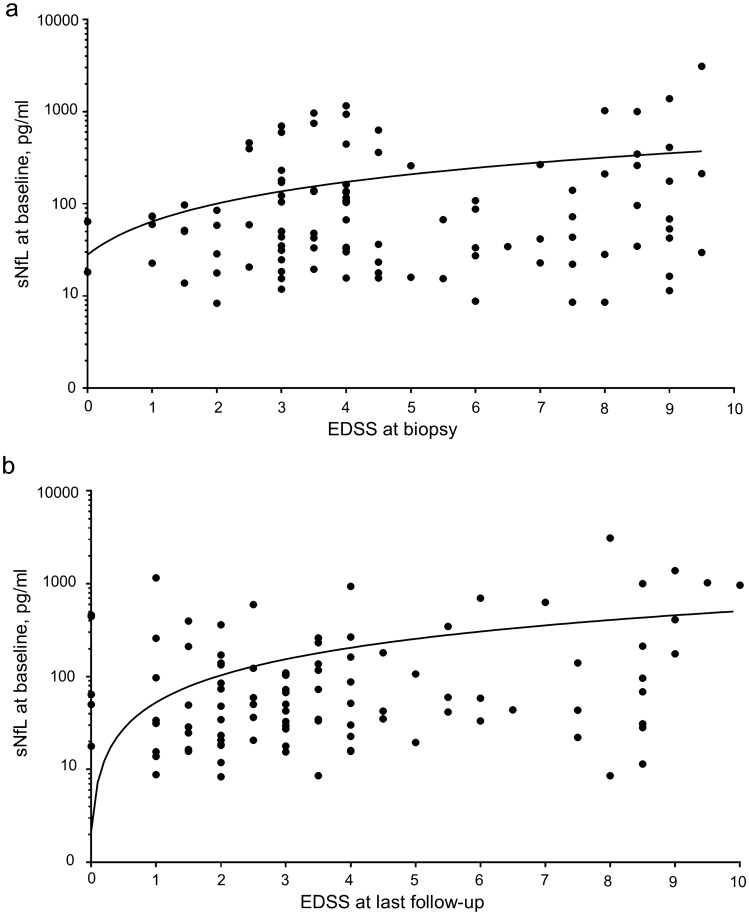
Correlation of sNfL levels at baseline with the EDSS at biopsy and at last follow-up. **a** A positive correlation between sNfL levels as the dependent variable and the EDSS at biopsy (independent variable) was found (*p* = 0.02). **b** sNfL levels correlate with the EDSS score at last follow-up (*p* < 0.001). A logarithmic scale was chosen for sNfL levels. *Bx* biopsy, *EDSS* expanded disability status scale

Finally, we correlated sNfL levels from the baseline blood sampling with clinical disability as measured with the EDSS. We found a positive correlation between sNfL levels and the EDSS at the time of biopsy (*p* = 0.02, Fig. 5a) as well as with the EDSS at 1 year (*p* < 0.01, not shown) and at last follow-up (*p* < 0.001; Fig. 5b).

## Discussion

The aim of our study was to perform histological–serological correlative analyses to determine whether axonal damage shown in histopathological sections corresponds to sNfL measured in patients with early MS. NfL are structural proteins of axons, and their detection in serum samples indicates axonal damage. However, sNfL are unspecific for the cause of the axonal damage, meaning that other diseases such as strokes or age-dependent degenerative processes may also elevate sNfL levels. Moreover, the exact origin within the CNS or even PNS of sNfL is still unknown. In MS patients, axonal damage may occur in newly forming as well as older lesions or in the normal-appearing white matter or the grey matter; the contribution of each to NfL levels in peripheral blood is not clear. We now provide evidence that the axonal damage found in earliest lesion stages as well as in the normal-appearing white matter correlates with NfL levels in serum.

A recent study by van den Bosch et al. focused on late-stage MS and correlated NfL in CSF from autopsied MS patients with histological parameters [48]. In our study, we established the correlation between NfL measured in serum and neuropathologic changes, since serum measurements would be easier to implement clinically than CSF measurements. Van den Bosch et al. found higher NfL levels in patients that showed lesion areas with foamy microglia/macrophages, and these lesions areas had higher numbers of acutely damaged axons compared to lesion areas without foamy microglia/macrophages [48]. While their study included mostly patients with progressive MS, our study complements their findings by focusing on MS patients with early MS and mostly monophasic and relapsing–remitting disease courses.

Previous histological analyses indicated that acute axonal damage is most pronounced in early MS lesion stages [5, 29], which was confirmed in our present study (Fig. 2a). Later lesion stages, the periplaque white matter and normal-appearing white matter also show acutely damaged axons, but the numbers of APP-positive spheroids are lower (Fig. 2a) [25, 29]. Our findings now demonstrate that sNfL levels in baseline samples directly correlate with acute axonal damage in the earliest lesion stages (Fig. 4a). This correlation may, on the one hand, be explained by a direct release of NfL from the newly formed lesions that led to biopsy. However, an elevation of sNfL normalizes within 9 months after brain injury [3], and only 48% of baseline blood samples were taken within 9 months after biopsy (Online Resource 1). Thus, on the other hand, it seems likely that the biopsy findings characterize the overall disease course of the patient, meaning that a patient with pronounced acute axonal damage at biopsy will be prone to axonal damage at follow-up. Evidence for this assumption comes from a large study investigating 589 patients with relapsing–remitting MS which showed that the strongest prognostic factor of high sNfL at the end of the study was a high sNfL level at baseline (*p* < 0.0001), suggesting an individual susceptibility for axonal damage [28].

There are multiple factors that influence sNfL levels. Previous studies have demonstrated that sNfL levels correlated with patient age, prior relapses as well as disease course [14, 28, 31], and our study confirmed these data for patient age and prior relapses (Table 2). We found a negative correlation between sNfL levels and the time interval after biopsy. No gender correlation was found in our or previous studies [14, 26]. Although there have been reports which demonstrated a decrease in sNfL during therapy [13, 28, 42], we found higher sNfL levels in patients treated either with immunomodulatory/immunosuppressive therapies or steroids. We assume that this finding reflects the more severe disease course (with higher sNfL levels) as the reason for initiating treatment, and that the treatment had not yet shown any effects. Longitudinal analysis of blood samples from individual patients are required to demonstrate treatment effects on sNFL levels.

The biopsy itself could cause high sNfL levels at baseline blood sampling as this procedure does constitute a brain injury. Even interventions such as positioning of intrathecal catheters or lumbar punctures were shown to increase sNfL levels [3]. In the present study, blood samples before and after biopsy were not available, so we were not able to directly determine the effect of the biopsy on sNfL levels. However, we could compare sNfL levels in patients who underwent stereotactic biopsy, which is considered to be less traumatic, with the potentially more destructive open surgery. sNfL levels in these cohorts did not differ significantly, making it less likely that the biopsy itself had a major influence on sNfL levels, although that cannot be completely ruled out.

Patients in our cohort showed initially very high sNfL levels (median 58.59 pg/ml at baseline blood sampling). In comparison, other studies reported concentrations between 10.1 and 32.9 pg/ml in patients with relapsing–remitting MS or a clinically isolated syndrome (CIS) [1, 6, 28]. High sNfL levels may reflect that this study investigated a special cohort of patients with acute and severe MS exacerbations that led to biopsy. As sNfL levels vary depending on the test applied, direct comparison of results must be done with caution [26]. However, the sNfL levels of healthy controls in our study were lower than those measured in previous studies (11.03 pg/ml versus 23.6 pg/ml), suggesting that our array does not measure higher levels in general [2]. Follow-up samples showed sNfL levels (median 21 pg/ml) similar to published cohorts, which most likely reflected the typical MS disease course of biopsied MS patients observed during follow-up [33, 34, 39].

Although sNfL levels were lower in the follow-up samples, they did not reach the level of the control samples, which thus indicates ongoing axonal damage. Only one patient had a relapse within 6 months prior to the follow-up blood sampling, which suggests that ongoing axonal damage occurs independent of relapses.

Prior histological studies have demonstrated that permanent axonal loss accumulates over time in MS patients, with the highest axonal loss within lesions [5, 7, 30]. In patients with long-standing MS, there are up to 80% fewer axons in spinal cord lesions than in normal-appearing white matter [7]. Normal-appearing white matter also undergoes pathological processes which may explain some of the chronic clinical disabilities [30]. Reports indicate up to 57% axonal loss in normal-appearing white matter of MS patients compared to healthy controls [7, 15, 16, 32]. Dziedzic et al. showed an axonal reduction of 7.2% in the periplaque white matter of biopsied patients, defined as non-demyelinated tissue surrounding MS lesions, in comparison to healthy controls [15]. This axonal loss may occur due to direct damage to axons or to Wallerian degeneration, i.e., the anterograde degeneration of the distal part of the axon that is damaged within MS lesions [4, 15].

To analyze the axonal damage that is not related to acute relapses, we correlated the sNfL levels in follow-up samples with axonal loss in lesions as well as in normal-appearing white matter. Our aim was to better understand the source of permanently elevated sNfL levels. When using simple regression analyses, a correlation between axonal density in the normal-appearing white matter and sNfL levels was found. These results suggest that an ongoing axonal loss in the normal-appearing white matter contributes to permanently elevated sNfL levels, which emphasizes the importance of the pathology of the normal-appearing white matter for axonal damage and clinical disability. Our data complement the findings of the recent study with histopathological–serological correlations in late-stage MS mentioned above: van den Bosch et al. also report that CSF NfL levels were positively correlated with increased axonal loss and with acute axonal damage in the normal-appearing white matter [48].

Imaging studies underline the relevance of pathological changes in the normal-appearing white matter and their correlation with sNfL. N-acetylaspartate MR spectroscopy revealed that MS patients with early disease stages and a low demyelinating lesion load had a reduced axonal density in the normal-appearing white matter, which indicates an axonal loss at least partially independent of lesions [12]. Furthermore, higher sNfL levels correlated with diffusion tensor imaging measurements in the normal-appearing white matter, which reflect diffuse microstructural damage and lower axonal density [41].

Importantly, sNfL parameters in the present investigation and in previous studies were found to correlate with clinical disability, which points to the clinical significance of axonal damage [10, 14]. The results in our biopsied cohort once again underscore that acute axonal damage in early lesion and disease stages is responsible for permanent clinical disability.

Our biopsied MS cohort provides a unique opportunity to carry out correlative histological–serological-clinical studies. Most of our biopsied patients are characterized by an atypical clinical presentation such as tumefactive lesions, which may be considered a limitation of this study. However, several previous studies have shown that results from biopsied patients can be extrapolated to prototypic MS, since the patients had a typical disease course during follow-up [33, 34, 39]. Another potential limitation of the present study is that the effect of the biopsy procedure itself on sNfL levels is not known. The procedure may increase sNfL levels, although we could not find any differences in sNfL levels between open surgery and stereotactic biopsy. An artificial elevation of sNfL levels could obscure correlations with histopathological findings, which we thus may have missed. Pre-biopsy sera were not available, since the patients were included in the study after their diagnosis of MS had been based on histopathological results.

The present study focused on early MS disease stages and complements histological–serological correlations in long-standing, progressive MS [48]. In early MS, clinical relapses and newly forming, gadolinium-enhancing lesions prevail, and we could show the correlation between acute axonal damage in earliest lesions stages and sNfL levels [20]. In late-stage progressive MS, active lesions are less frequent but when present, they also correlate with higher sNfL levels [20, 48]. More pronounced changes in the normal-appearing white matter can be seen in late-stage progressive MS compared to relapsing–remitting MS [22, 30]. Nonetheless, our study now shows that not only in progressive MS but already in early disease stages, sNfL levels correlated with axonal damage in the normal-appearing white matter [48]. The contribution of cortical lesions is still unknown and thus should be analyzed in future studies.

## Conclusions

Our study demonstrates that sNfL levels correlate with acute axonal damage in earliest MS lesion stages, suggesting that sNfL represent a compound measure of recent and ongoing neuroaxonal damage [28]. Measurement of sNfL levels thus allows insight into brain pathology, and our results underline the relevance of relapse-associated lesional pathology for axonal damage in early MS. Independent of relapses, axonal loss in normal-appearing white matter additionally seems to contribute to sNfL levels, which also points to the clinical importance of pathological changes in normal-appearing white matter. Axonal damage correlates with clinical disability, and measurement of sNfL levels may serve as an important biomarker to help clinicians make better individual treatment decisions.

## Supplementary Information

Below is the link to the electronic supplementary material.

Online Resource 1: Time interval between biopsy and baseline blood sampling. The time interval between the biopsy and the first (baseline) blood sampling is shown for the 106 study participants. Note the varying time intervals that were chosen (before biopsy, 3 months intervals for 0–12 months, yearly intervals for 1–8 years and single time points thereafter). (JPG 109 KB)

Online Resource 2: SNfL group comparisons. **a** Group comparison of sNfL levels in patients with and without a relapse within six months before baseline blood sampling. Patients with a relapse within six months prior to blood sampling showed significantly higher sNfL levels than patients without a relapse (*p*<0.001). The median, 25th and 75th percentiles are given. **b** Group comparison of sNfL levels in patients with and without steroid treatment within six weeks before baseline blood sampling. Patients treated with steroids within six weeks prior to blood sampling showed significantly higher sNfL levels than patients with no steroid treatment (*p*<0.01). The median, 25th and 75th percentiles are given. (TIF 564 KB)

Online Resource 3: Correlation of sNfL levels of the follow-up blood samples and axonal density in the normal-appearing white matter. A negative correlation (*p*=0.02) between sNfL levels at follow-up and axonal density in the normal-appearing white matter (NAWM) was found. The dependent variable is the sNfL level and the independent variable is relative axonal density (the number of axons crossing the stereological grid’s point from a total number of 25 grid points). (TIF 249 KB)

## Data Availability

Data are available from the authors upon reasonable request.
