# Supplementary figures and images for: Neurofilament light chains in serum as biomarkers of axonal damage in early MS lesions: a histological–serological correlative study
Source: J Neurol. 2022 Nov 13;270(3):1416–29. doi: 10.1007/s00415-022-11468-2 (PMC9971126; doi:10.1007/s00415-022-11468-2)

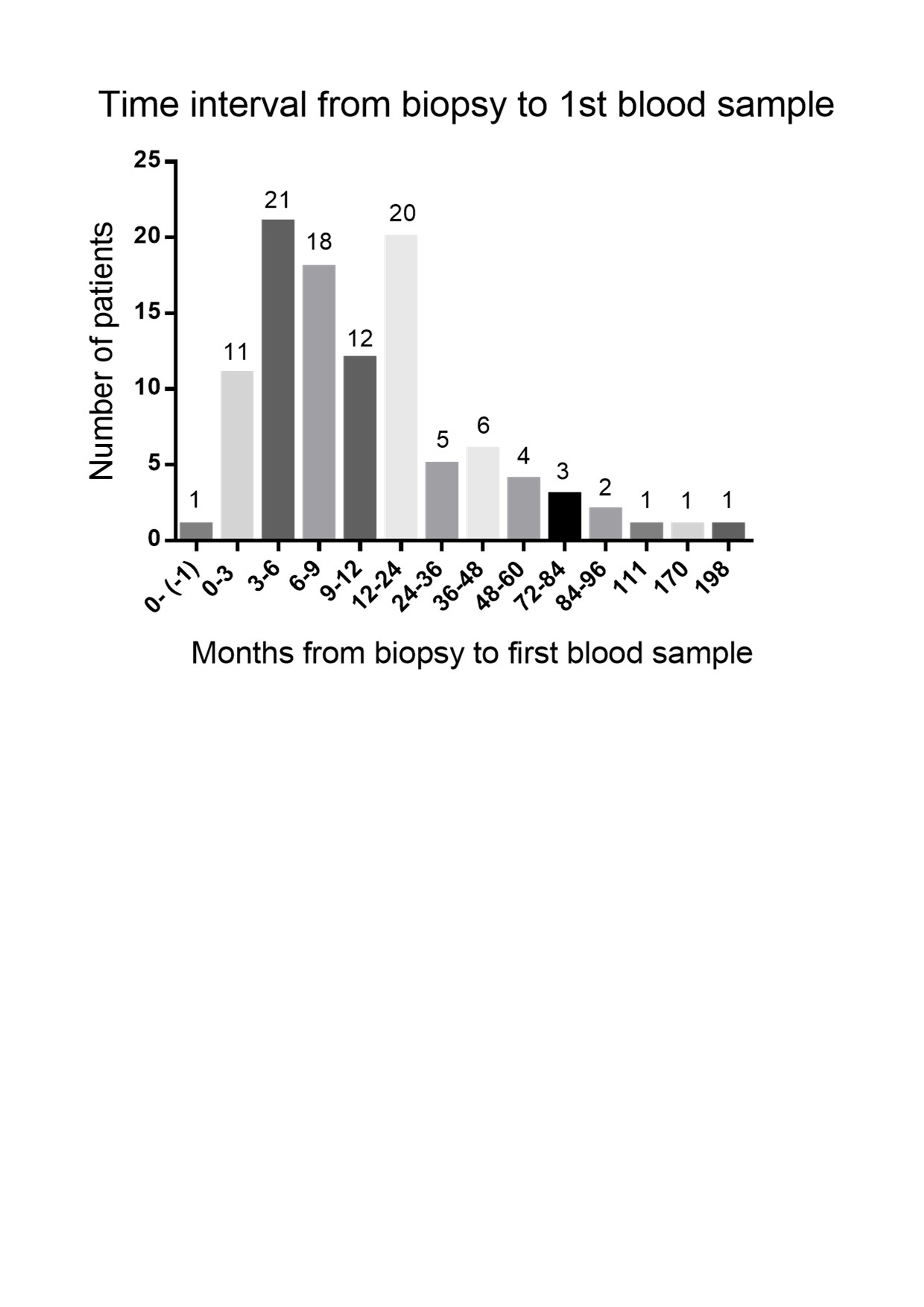

Supplement: Supplementary file 1 — Online Resource 1: Time interval between biopsy and baseline blood sampling. The time interval between the biopsy and the first (baseline) blood sampling is shown for the 106 study participants. Note the varying time intervals that were chosen (before biopsy, 3 months intervals for 0–12 months, yearly intervals for 1–8 years and single time points thereafter). (JPG 109 KB) [file 415_2022_11468_MOESM1_ESM.jpg]

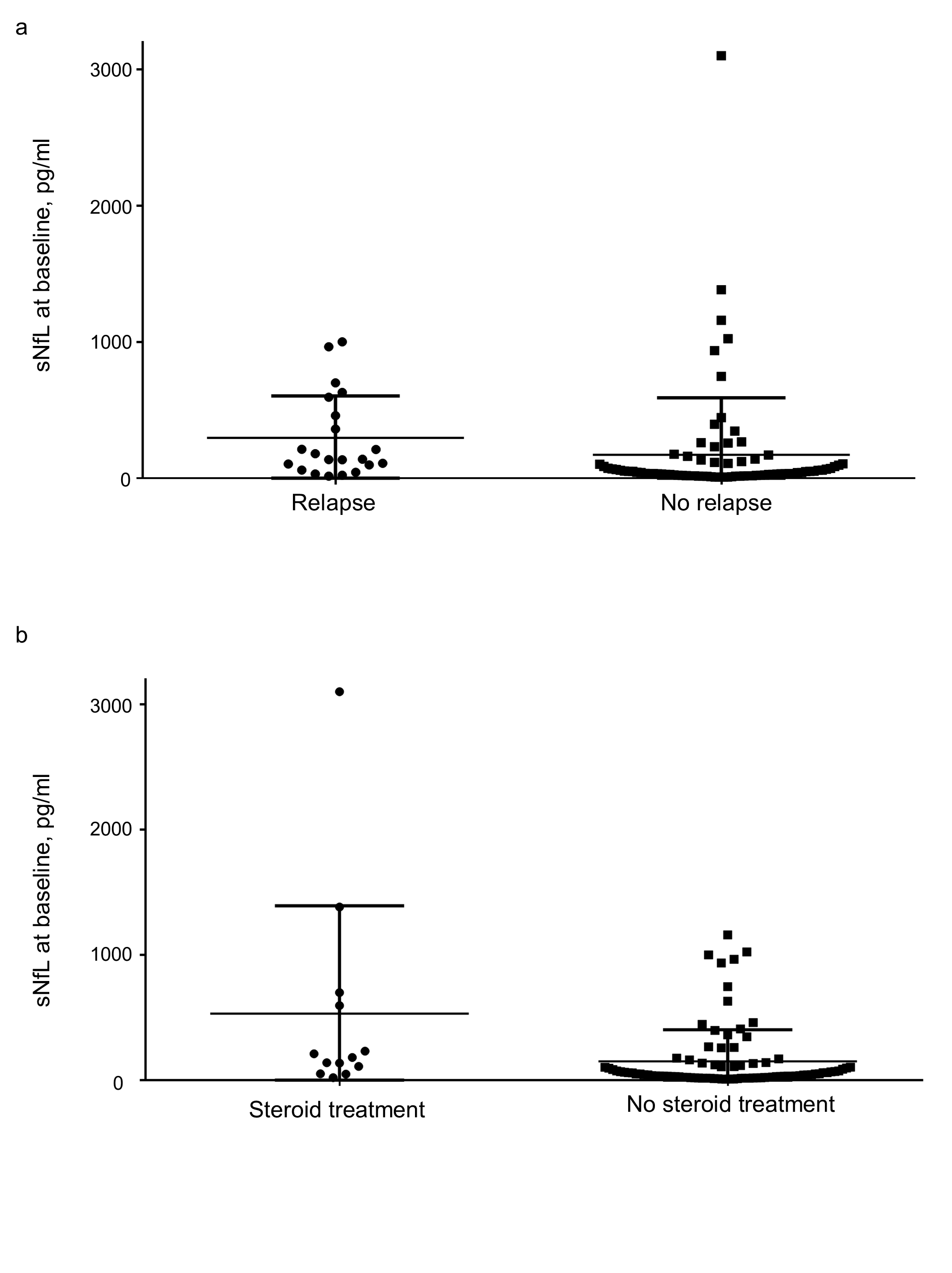

Supplement: Supplementary file 2 — Online Resource 2: SNfL group comparisons. a Group comparison of sNfL levels in patients with and without a relapse within six months before baseline blood sampling. Patients with a relapse within six months prior to blood sampling showed significantly higher sNfL levels than patients without a relapse (p<0.001). The median, 25th and 75th percentiles are given. b Group comparison of sNfL levels in patients with and without steroid treatment within six weeks before baseline blood sampling. Patients treated with steroids within six weeks prior to blood sampling showed significantly higher sNfL levels than patients with no steroid treatment (p<0.01). The median, 25th and 75th percentiles are given. (TIF 564 KB) [file 415_2022_11468_MOESM2_ESM.tif]

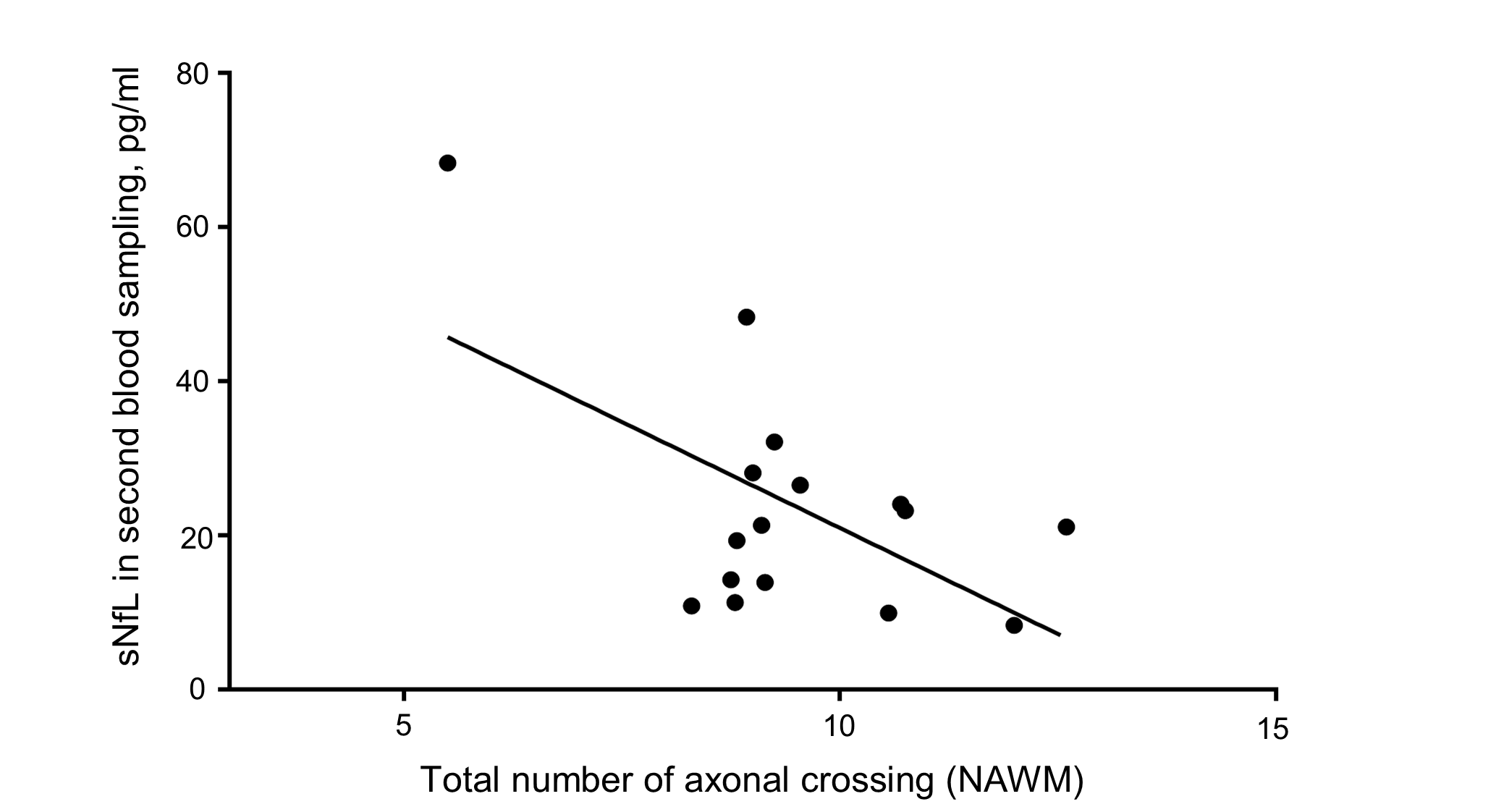

Supplement: Supplementary file 3 — Online Resource 3: Correlation of sNfL levels of the follow-up blood samples and axonal density in the normal-appearing white matter. A negative correlation (p=0.02) between sNfL levels at follow-up and axonal density in the normal-appearing white matter (NAWM) was found. The dependent variable is the sNfL level and the independent variable is relative axonal density (the number of axons crossing the stereological grid’s point from a total number of 25 grid points). (TIF 249 KB) [file 415_2022_11468_MOESM3_ESM.tif]
